# A cost-effectiveness analysis of artemether lumefantrine for treatment of uncomplicated malaria in Zambia

**DOI:** 10.1186/1475-2875-6-21

**Published:** 2007-02-21

**Authors:** Pascalina Chanda, Felix Masiye, Bona M Chitah, Naawa Sipilanyambe, Moonga Hawela, Patrick Banda, Tuoyo Okorosobo

**Affiliations:** 1 National Malaria Control Centre, Box 32 509, Lusaka, Zambia; 2 Department of Economics, University of Zambia, Lusaka, Zambia; 3 Ministry of Health, Lusaka, Zambia; 4 Regional Office for Africa, World Health Organization, Cité du Djoué, Brazzaville, Congo

## Abstract

**Background:**

Malaria remains a leading cause of morbidity, mortality and non-fatal disability in Zambia, especially among children, pregnant women and the poor. Data gathered by the National Malaria Control Centre has shown that recently observed widespread treatment failure of SP and chloroquine precipitated a surge in malaria-related morbidity and mortality. As a result, the Government has recently replaced chloroquine and SP with combination therapy as first-line treatment for malaria. Despite the acclaimed therapeutic advantages of ACTs over monotherapies with SP and CQ, the cost of ACTs is much greater, raising concerns about affordability in many  poor countries such as Zambia. This study evaluates the cost-effectiveness analysis of  artemether-lumefantrine, a version of ACTs adopted in Zambia in mid 2004.

**Methods:**

Using data gathered from patients presenting at public health facilities with suspected malaria, the costs and effects of using ACTs versus SP as first-line treatment for malaria were estimated. The study was conducted in six district sites. Treatment success and  reduction in demand for second line treatment constituted the main effectiveness outcomes. The study gathered data on the efficacy of, and compliance to,  AL and SP treatment from a random sample of patients. Costs are based on estimated drug, labour, operational  and capital inputs. Drug costs were based on dosages and unit prices provided by the Ministry of Health and the manufacturer (Norvatis).

**Findings:**

The results suggest that AL produces successful treatment at less cost than SP, implying that AL is  more cost-effective. While it is acknowledged that  implementing national ACT program will require considerable resources, the study demonstrates that the health gains (treatment success) from every dollar spent are significantly greater if AL is used rather than SP. The incremental cost-effectiveness ratio is estimated to be US$4.10. When the costs of second line treatment are considered the ICER of AL becomes negative, indicating that there are greater resource savings associated with AL in terms of reduction of costs of complicated malaria treatment.

**Conclusion:**

This study suggests the decision to adopt AL is justifiable on both economic and public health  grounds.

## Background

Malaria remains a leading cause of morbidity, mortality and non-fatal disability in Zambia, especially among children, pregnant women and the poor. The disease burden caused by malaria in Zambia has grown steadily over the recent decades. Malaria is endemic in most parts of Zambia although rural areas and poor urban cities tend to bear a disproportionate share of malaria transmission and burden. Estimates based on the Health Information System (HIS) suggest that malaria incidence has increased from 121.5 per 1,000 in 1976 to 429.3 per 1000 in 2003 [1,2]. Recent statistics show that in 2003 some 3.5 million malaria cases were attended to at public health facilities. In the same year, malaria accounted for 23% of all deaths occurring at hospitals, making it the leading cause of death in the country [1,3]. Malaria, especially in its severe form, affects people in more ways than these metrics can measure. For example, malaria is known to impair the general immunity of children, leaving them susceptible to other causes of illness and death. Malaria also affects the cognitive ability of individuals [4]. The difficulties with the validity of these numbers notwithstanding, malaria is still considered to be a  major health problem that affects the widest section of the Zambian population.

Over the recent past, health information and various surveys have revealed that widespread treatment failure precipitated a rise in malaria mortality and morbidity. National data collected by the National Malaria Control Centre (NMCC) confirmed that a considerable decline in the therapeutic efficacy of sulphadoxine-pyrimethamine (SP) and chloroquine (CQ) was responsible for the high and widespread treatment failure rates [5]. This situation had significant implications since treatment with anti-malarial drugs has been the only tool used in fighting malaria from the late 1970s. In terms of prospects for young children, most of whom are treated at home, failure of the only possible defence against malaria meant rising mortality.

Widespread treatment failure was an Africa-wide phenomenon which was observed from the late 1980's and spread rapidly from then on. The evidence from several studies pointed unequivocally to growing drug resistance and childhood mortality [6]. In response to growing criticism against the use of failing monotherapies, the World Health Organization and Roll-Back Malaria led a global campaign to replace SP and chloroquine with artemesinin combination therapies (ACT) as first-line treatment [7]. To date, not all countries in Africa have implemented ACTs as first-line treatment for malaria. The two most widely considered ACT brands in Africa presently are the fixed-dose combination artemether-lumefantrine (AL) and the co-packaged combination of amodiaquine and artesunate (AQ+AS). These combinations have proved highly efficacious in carefully controlled phase III clinical trials in areas with moderate to high levels of SP or CQ resistance in Africa [7].

In 2004, Zambia adopted AL as the new first-line drug for treatment of malaria in all public health facilities. The policy pronouncement was informed by the widely acclaimed efficacy of ACTs without a comprehensive economic evaluation. From a therapeutic point of view artemesinin-based products have been shown to have important advantages: if they are used in combination with other anti-malarial therapies, the rate at which resistance emerges will be slowed down. In addition, ACTs clear parasites faster and more completely than classical drugs [8,9]. In this way, they reduce treatment failure and a recurrence of new malaria episodes thereby reducing the probability of progression to severe malaria. In this way, ACTs, avert malaria mortality and various forms of debilitating sequelae that are associated with episodes of severe malaria especially on children [8]. Unfortunately, ACTs are significantly more expensive than the existing anti-malarials. Thus, from an economic point of view, the question arises if the therapeutic benefits of ACTs outweigh the extra cost of ACTs. This economic framework embodies in cost-effectiveness analysis addresses the question of whether, given a fixed budget, the Zambian health care system could achieve better health gains with ACTs or with the existing anti-malarial drugs, namely SP.

One of the main concerns with scaling up implementation ACTs in Zambia and other African countries has been the cost [10]. Given the difficult trade-offs that the Zambian government faces in terms of allocating resources across a broad disease spectrum, effectiveness alone is unlikely to be a sufficient premise for initiating a new intervention. In this particular case, given the high cost of, a greater need arises to ensure that there is efficiency and effectiveness in the diagnosis of malaria and its treatment with the usage of Artemether-lumefantrine. In Zambia, an intense national debate emerged in the wake of this new policy. Opponents and pessimists doubted the cost-effectiveness of ACTs given their high prices and also the weaknesses in the diagnostic and prescribing capacities of the health system. This study sought to generate systematic evidence on the clinical effects, cost and cost-effectiveness of AL from an actual practice routine malaria management framework. The purpose of this survey was to assess the cost-effectiveness of AL in reference to SP for the treatment of uncomplicated malaria.

## Methods and data

This study was designed to capture data on health outcomes from a real health setting. Rather than employ a randomized control trial approach, the study employed data generated by the health system through its routine systems. The main rationale for taking this approach was that it is unlikely that even the introduction of ACTs was going to interfere with the current treatment protocol which requires presumptive treatment of most febrile cases in children. In Zambia, this practice is often extended to adults. Treatment practices clearly affect the cost-effectiveness of ACTs. In this context, it is considered crucial to test a cost-effectiveness model that is based on data generated from actual practice. Another important factor to consider for cost-effectiveness analysis is that AL in Zambia was introduced in a context of very low coverage of prevention strategies. According to the 2001/2 Demographic and Health Survey (DHS), the national coverage of insecticide treated bed-nets for children under the age of five years in Zambia is now only 23% after a mass distribution campaign that took place after the period of this survey [11]. Therefore, the cost-effectiveness analysis conducted here should be interpreted in this context that the underlying incidence of malaria in the population is unlikely to have changed significantly. Nonetheless, the main purpose was to demonstrate the cost-effectiveness of AL as a first-line drug.

### Study design and population

This study was designed around building a data base capturing the short-term effects and cost of treating patients with a new malaria drug. Thus, the study population included all patients presenting with suspected malaria visiting outpatient facilities (health centres). To achieve this, health staff at the study sites were thoroughly trained to record data on diagnosis and treatment from all patients suspected of having malaria into a specially-designed register. The data on diagnosis, temperature, treatment provided, outcome within 28 days, follow up action and some basic patient characteristics were recorded into these registers. Consistent with the perspective taken, malaria was defined using the standard Ministry of Health Malaria Case Management Manual as operationalised by health workers. Further, Data on all the malaria attendances from the period 2000 to 2004 (so-called retrospective data) were retrieved from registers at the study facilities. These represented both confirmed and non-confirmed cases.

Ideally, all patients are requested to report for a review 10 days after treatment to ascertain their response to treatment. However, less than 10% of the patients turned up, implying that only a survey-based method would yield valid and reliable data on the therapeutic effects of AL and associated patient compliance. Thus, data on treatment outcome and patient compliance within a 28-day period were compiled in a follow-up survey on a sample from the same population that was conducted during the same time period. During these surveys, parasitological confirmation of cure was performed using microscopy. Standard WHO protocols were followed in conducting the efficacy of anti-malarials as well as compliance surveys. Efficacy and compliance surveys were handled by an independent team from the National Malaria Control Centre to ensure validity. Details of the study and protocol are available at the National Malaria Control Centre. Full details on the procedure of efficacy and compliance determination have been published elsewhere [12-14]. Finally, previous evaluation surveys [15,16] provided the applicable Zambian estimates for efficacy and compliance to the previous anti-malarial regimens (SP) in Zambia.

### Survey administration and data handling

A total of six study districts were selected by the NMCC for the study; Kabwe, Chongwe, Chipata, Kalomo, Chingola, and Samfya. These districts are part of the ten Roll-Back Malaria (RBM) monitoring sentinel surveillance sites. They were chosen based on geographical distribution and the malaria incidence strata. In each district, three study sites (health facility) were purposively sampled. Three facilities from each district were selected and assigned one of each type of malaria diagnosis (microscopy, rapid diagnostic tests or clinical) depending on current practice. The existing microscopy services at some health facility were maintained while RDTs were introduced in facilities with no diagnostic services. This brought the total of facilities under investigation to 18 (3 facilities from each district).

After the sites were selected, initial orientation of health staff was conducted on data capturing, record keeping and the need to observe and record review cases. The person in-charge of each facility was responsible for supervising the in-house data collection process. Further, within each district, there is a malaria information officer who provided weekly supervisory visits to three facilities under their jurisdiction. The national team was also responsible for close supervision, on a monthly basis to ensure that supplies were adequate and that record keeping was being adhered to. During these visits, data entry was scrutinized to ensure that register scores matched tally sheets. Members of staff were also interviewed on how the survey was proceeding. The three levels of supervision helped to ensure that data collection instructions were strictly followed.

Data entry was undertaken in a Microsoft Access program based data base through customized data entry screens with in-built range and consistency checks. The data was entered by independent data entry clerks, with completed data files compared for data-entry errors. All data entry and management was undertaken at the National Malaria Control Centre (NMCC). Data entry was closely supervised with any summaries generated from the database compared with hand-tallied summaries to ensure consistency. Data collection proceeded from February through November 2005.

#### Effectiveness indicators

The clinical impact of AL is usually considered in terms of: (i) prevention of treatment failure associated with SP, and (ii) prevention of aggravation from simple malaria to severe malaria. In line with these attributes, the study defined two indicators of effectiveness. The first indicator considered the number of cases of malaria successfully treated. This indicator assumes that the main aim of treatment policy is to maximize the number of cases successfully treated from available resources. In the study design, successful treatment was defined as patient showing negative parasitaemia after treatment with AL. This measure was computed from field data on efficacy, compliance and clinical success from a paired survey on the same patient population. A simple model was constructed to predict the number of successfully treated cases under the two treatment regimens.

The second indicator of clinical effectiveness was the proportion of malaria cases proceeding to severe malaria, which essentially indicates the degree to which AL prevents disease aggravation [17]. Severe malaria is also a good predictor of malaria mortality. According to case management guidelines, severe malaria is identified by any of several typical clinical syndromes including: high fever with respiratory distress (abnormally fast breathing), convulsions, hyperparasitaemia (where microscopy is available), anaemic symptoms and neurological disturbances. This indicator of effectiveness was mirrored in as health outcome data namely, number of clinical severe malaria cases as a proportion of uncomplicated malaria cases. Severe malaria cases data was collected also from the facility registers from all the 18 facilities under study during the entire study period.

### Costing

The costing was done from a providers' perspective. (i.e. the public health system). This means that only costs borne by the health facilities during the provision of malaria treatment services have been considered. Cost elements included were drugs, personnel, medical examination, materials, administrative and equipment overheads, and building space. Except for drug and medical examination costs, all other costs were treated as overheads with the malaria-related cost derived using the direct attribution method (i.e. relative malaria prevalence). After obtaining cost attributed to malaria, average total cost per malaria case was calculated as the total malaria related costs divided by total number of malaria cases. Cost data were gathered from both secondary and primary sources.

A questionnaire was designed and used to capture data from health facilities on quantities and prices of various cost inputs including capital assets, equipment, materials and personnel. Capital assets typically included building space in meters, motorbikes, motor vehicles and furniture such as chairs, tables and cabinets. Facilities reported only basic equipment such as microscope, stethoscope, sphygmomanometer, thermometer, laboratory equipment, refrigerator, cold chain, weighing scales. Purchase prices obtained from health facilities were used to derive discounted annual capital costs. Equipment and motor vehicles were annualized using a discount rate of 3% and lifespan of five years, while buildings were given a life span of 30 years at the same discount rate. The 3% discount rate has been used extensively in the literature [18].

Data on the cost per square meter of health facility was provided by the Physical Planning Division of the Ministry of Health. Once the costing was done, allocation of costs attributable to malaria was based on total utilization of OPD resources for other diseases as a denominator and total malaria utilization as the numerator. It has been assumed that 40% of OPD cases are due to malaria.

Labour costs were computed using the time spent by each staff cadre in treating a case of malaria or suspected malaria. Staff time was multiplied by pro rata earnings (salary plus standard allowances) for each staff category involved in treating malaria. Salary data was gathered from respective staff members. All cost estimates from past years was converted to 2005 prices using official exchange rate US$1 = ZMK3,500. The costs were computed using standard costing approaches in economic evaluation [18]. Costs of switching to other forms of treatment during a single illness episode have not been included in this costing.

### Cost of second-line treatment

In addition, this study required estimating the cost of treating patients with complicated or mildly severe malaria. An investigation of actual practice for patients not cured within the 28-day period revealed the following scenarios: (i) three waves of treatment are administered to patients failing first line treatment (ii) returning patients receive repeated first-line treatment regardless of parasitological status, (iii) efficacy of repeated first-line unknown, and (iv) after failure of second attempt, returning patients are either referred further or treated with second-line treatment. This implies that after repeated first-line fails, patients either develop complicated malaria, get cured, seek alternative providers (including home remedies) or are treated for something else. With the exception of the first, the potential effects of these possibilities in an incremental framework are held constant in this study. This is a strong assumption. According to the data, 4.7% of AL recipients and 48.1% of SP recipients were treated for complicated malaria. Further, it was assumed that the efficacy of second-line treatment is the same regardless of first-line treatment given. These scenarios were constructed using data obtained from malaria facility registers, field surveys and interviews with health workers.

The methodology of costing described above was used to obtain the average cost of treating an episode of complicated malaria with second line treatment. However, complicated malaria is treated at various levels within the health care system, with each of these levels having different costs. Patients with complicated malaria present themselves at four facility options namely health centre outpatient, health centre inpatient, hospital outpatient and hospital admission. Data on the fraction of patients seeking severe malaria treatment at each of these facilities as well as estimated treatment costs for complicated malaria treatment were obtained from two recent surveys [19,20]. The cost per case of severe malaria treated was calculated using as a weighted average of the cost per episode at four different levels.

### Methods of analysis

The cost-effectiveness model used considers three important elements. First, the study reports on estimates of the treatment cost per episode. Treatment cost measures and values the resource inputs that are required to produce a malaria therapy. Second, the study estimates the average cost-effectiveness ratio (ACER). The ACER estimates the cost per each case of malaria success fully treated. Formally, this is defined as the total cost of treating all cases somehow diagnosed as malaria divided by the total number of cases considered having been cured within the 28-day period. This means that, holding cost constant, a therapy with the highest number of successful treatment outcomes will have a lower ACER. The lower the ACER the more preferred the therapy.

The formula of the average cost-effectiveness ratios is as follows,

ACERAL=Estimated total cost of treatment (AL)Effectiveness (AL),
 MathType@MTEF@5@5@+=feaafiart1ev1aaatCvAUfKttLearuWrP9MDH5MBPbIqV92AaeXatLxBI9gBaebbnrfifHhDYfgasaacH8akY=wiFfYdH8Gipec8Eeeu0xXdbba9frFj0=OqFfea0dXdd9vqai=hGuQ8kuc9pgc9s8qqaq=dirpe0xb9q8qiLsFr0=vr0=vr0dc8meaabaqaciaacaGaaeqabaqabeGadaaakeaacqWGbbqqcqWGdbWqcqWGfbqrcqWGsbGudaWgaaWcbaGaemyqaeKaemitaWeabeaakiabg2da9maalaaabaGaeeyrauKaee4CamNaeeiDaqNaeeyAaKMaeeyBa0MaeeyyaeMaeeiDaqNaeeyzauMaeeizaqMaeeiiaaIaeeiDaqNaee4Ba8MaeeiDaqNaeeyyaeMaeeiBaWMaeeiiaaIaee4yamMaee4Ba8Maee4CamNaeeiDaqNaeeiiaaIaee4Ba8MaeeOzayMaeeiiaaIaeeiDaqNaeeOCaiNaeeyzauMaeeyyaeMaeeiDaqNaeeyBa0MaeeyzauMaeeOBa4MaeeiDaqNaeeiiaaIaeiikaGIaeeyqaeKaeeitaWKaeiykaKcabaGaeeyrauKaeeOzayMaeeOzayMaeeyzauMaee4yamMaeeiDaqNaeeyAaKMaeeODayNaeeyzauMaeeOBa4MaeeyzauMaee4CamNaee4CamNaeeiiaaIaeeikaGIaeeyqaeKaeeitaWKaeeykaKcaaiabcYcaSaaa@7AC0@

ACERSP=Estimated total cost of treatment (SP)Effectiveness (SP).
 MathType@MTEF@5@5@+=feaafiart1ev1aaatCvAUfKttLearuWrP9MDH5MBPbIqV92AaeXatLxBI9gBaebbnrfifHhDYfgasaacH8akY=wiFfYdH8Gipec8Eeeu0xXdbba9frFj0=OqFfea0dXdd9vqai=hGuQ8kuc9pgc9s8qqaq=dirpe0xb9q8qiLsFr0=vr0=vr0dc8meaabaqaciaacaGaaeqabaqabeGadaaakeaacqWGbbqqcqWGdbWqcqWGfbqrcqWGsbGudaWgaaWcbaGaem4uamLaemiuaafabeaakiabg2da9maalaaabaGaeeyrauKaee4CamNaeeiDaqNaeeyAaKMaeeyBa0MaeeyyaeMaeeiDaqNaeeyzauMaeeizaqMaeeiiaaIaeeiDaqNaee4Ba8MaeeiDaqNaeeyyaeMaeeiBaWMaeeiiaaIaee4yamMaee4Ba8Maee4CamNaeeiDaqNaeeiiaaIaee4Ba8MaeeOzayMaeeiiaaIaeeiDaqNaeeOCaiNaeeyzauMaeeyyaeMaeeiDaqNaeeyBa0MaeeyzauMaeeOBa4MaeeiDaqNaeeiiaaIaeiikaGIaee4uamLaeeiuaaLaeiykaKcabaGaeeyrauKaeeOzayMaeeOzayMaeeyzauMaee4yamMaeeiDaqNaeeyAaKMaeeODayNaeeyzauMaeeOBa4MaeeyzauMaee4CamNaee4CamNaeeiiaaIaeeikaGIaee4uamLaeeiuaaLaeeykaKcaaiabc6caUaaa@7B48@

Finally, when thinking about a new alternative drug (AL), which would be mutually exclusive with an existing drug (SP), the amount of resources available becomes an important consideration. An appropriate framework for addressing this question is the incremental cost effectiveness ratio (ICER). The ICER measures the additional cost that is required in order to achieve a superior health effect over the baseline. For instance, a switch from SP to AL has meant that a more expensive drug is being used to compensate for the inferior health benefits of SP. Thus, the extra health benefits of AL come with an extra cost. Intuitively, the ICER is defined as the additional cost of treatment with AL divided by the additional health effects of AL. This analysis begs the question of whether the extra cost per additional benefit generated is worth paying for or not. Although this question is germane to a different form of economic evaluation known as cost-benefit analysis, or may be even to politics [18,21]. The idea is to compare these results with similar studies in similar countries for similar studies and also to refer to ICER thresholds that been customarily applied in cost-effectiveness studies.

The formula for calculating the ICER was defined as follows,

ICERAL,SP=Expected total cost of treatment (ACT) - Expected total cost of treatment (SP)Expected effectiveness (ACT) - Expected effectiveness (SP)
 MathType@MTEF@5@5@+=feaafiart1ev1aaatCvAUfKttLearuWrP9MDH5MBPbIqV92AaeXatLxBI9gBaebbnrfifHhDYfgasaacH8akY=wiFfYdH8Gipec8Eeeu0xXdbba9frFj0=OqFfea0dXdd9vqai=hGuQ8kuc9pgc9s8qqaq=dirpe0xb9q8qiLsFr0=vr0=vr0dc8meaabaqaciaacaGaaeqabaqabeGadaaakeaacqWGjbqscqWGdbWqcqWGfbqrcqWGsbGudaWgaaWcbaGaemyqaeKaemitaWKaeiilaWIaem4uamLaemiuaafabeaakiabg2da9maalaaabaGaeeyrauKaeeiEaGNaeeiCaaNaeeyzauMaee4yamMaeeiDaqNaeeyzauMaeeizaqMaeeiiaaIaeeiDaqNaee4Ba8MaeeiDaqNaeeyyaeMaeeiBaWMaeeiiaaIaee4yamMaee4Ba8Maee4CamNaeeiDaqNaeeiiaaIaee4Ba8MaeeOzayMaeeiiaaIaeeiDaqNaeeOCaiNaeeyzauMaeeyyaeMaeeiDaqNaeeyBa0MaeeyzauMaeeOBa4MaeeiDaqNaeeiiaaIaeeikaGIaeeyqaeKaee4qamKaeeivaqLaeeykaKIaeeiiaaIaeeyla0IaeeiiaaIaeeyrauKaeeiEaGNaeeiCaaNaeeyzauMaee4yamMaeeiDaqNaeeyzauMaeeizaqMaeeiiaaIaeeiDaqNaee4Ba8MaeeiDaqNaeeyyaeMaeeiBaWMaeeiiaaIaee4yamMaee4Ba8Maee4CamNaeeiDaqNaeeiiaaIaee4Ba8MaeeOzayMaeeiiaaIaeeiDaqNaeeOCaiNaeeyzauMaeeyyaeMaeeiDaqNaeeyBa0MaeeyzauMaeeOBa4MaeeiDaqNaeeiiaaIaeiikaGIaee4uamLaeeiuaaLaeiykaKcabaGaeeyrauKaeeiEaGNaeeiCaaNaeeyzauMaee4yamMaeeiDaqNaeeyzauMaeeizaqMaeeiiaaIaeeyzauMaeeOzayMaeeOzayMaeeyzauMaee4yamMaeeiDaqNaeeyAaKMaeeODayNaeeyzauMaeeOBa4MaeeyzauMaee4CamNaee4CamNaeeiiaaIaeeikaGIaeeyqaeKaee4qamKaeeivaqLaeeykaKIaeeiiaaIaeeyla0IaeeiiaaIaeeyrauKaeeiEaGNaeeiCaaNaeeyzauMaee4yamMaeeiDaqNaeeyzauMaeeizaqMaeeiiaaIaeeyzauMaeeOzayMaeeOzayMaeeyzauMaee4yamMaeeiDaqNaeeyAaKMaeeODayNaeeyzauMaeeOBa4MaeeyzauMaee4CamNaee4CamNaeeiiaaIaeeikaGIaee4uamLaeeiuaaLaeeykaKcaaaaa@DE78@

### Ethical considerations

This study was approved by the Ethics Committee at the Tropical Disease Research Centre (TDRC). The study was also cleared by the Director General's Office of the Central Board of Health. Further, local support was sought from the District Director of Health in each district and also the in-charge of the given facilities. Such consensus led to high cooperation in terms of the study operation and logistics. No names of patients were divulged during data analysis, only age and sex characteristics were shown. As the study data gathering process was restricted to malaria registers at facilities, with no contact with patients or patient names, no major ethical issues arose.

## Results

### Cost of treatment

Table 1 presents estimates of the average cost of treating an episode of uncomplicated malaria with SP compared with AL. Costs included drugs, personnel, medical examination, and administrative and equipment overheads and capital cost. Data for estimating costs was obtained from facilities. It is shown that the cost of treatment with SP (US$6.19) is far less than treatment with AL (US$7.34), implying that AL will increase the overall cost malaria treatment in Zambia. It can also be seen that the introduction of the more expensive AL changes the cost structure of the malaria programme by increasing the drug component of malaria budget of Ministry of Health. The average cost per case treated is almost 20% higher with AL. It can also be seen that the cost structure has changed drastically with the drug component of total direct health system costs of treating malaria having gone up six fold.

**Table 1:** Average costs of treating an episode of uncomplicated malaria: SP and AL

Cost item	AL		SP	
	Cost (US$)	percentage	Cost (US$)	Percentage

1. Drugs	1.33	18	0.18	3
2. Diagnostics (medical examination)	4.25	58	4.25	69
3. Personnel	0.84	12	0.84	14
4. Administration and other overheads compliance	0.54	7	0.54	9
5. Capital (equipment and buildings)	0.38	5	0.38	6
6. Average Total cost per patient treated	7.34	100	6.19	100

These estimates compare very well with comparable estimates from a Tanzanian study in which the cost of SP and AL were US$5.09 and US$5.96, respectively [22]. However, if converted to year 2000 international dollars, these estimates are nearly 50% higher than the estimates derived from the World Health Organisation cost-effectiveness model known as the WHO-CHOICE. The WHO-CHOICE cost estimate per episode was $5.97 in 2000 international (i.e. purchasing power parity) dollars excluding drugs and diagnostic components [23].

### Cost-effectiveness analyses

Using costs and effects estimated above, the cost-effectiveness ratios were estimated as depicted in table 2. According to the cost-effectiveness framework described earlier, the cost per case successfully treated as well as the incremental cost per each extra case successfully cured, with AL in relation to SP, were estimated.

**Table 2:** Estimated number of cases successfully treated and average cost-effectiveness

**Effects/cost**	**AL**	**SP**
1. Patients starting treatment (2005)	55509	55509
2. Efficacy	98.2%	68.4%
3. Patient Compliance	75.1%	85.0%
4. Treatment successful despite less than full compliance	50%	0
5. Number of cases successfully treated*	47560	32273
6. Total expenditure on treating malaria	406370.69	343743.64
7. Average Cost-effectiveness Ratio (average cost per case cured)	$8.57	$10.65
8. Incremental cost per case successfully treated	4.10	-

Items in Rows 5, 6, 7 and 8 estimated from formula in section 3. Input data on efficacy and compliance obtained from surveys [12-16].

### Cost-effectiveness ratio for cost per case successfully treated

The first element of the cost-effectiveness analysis was the average cost per case successfully treated patient (ACER): SP versus AL. A simple model illustrated in Table 2 has been used to derive treatment success and the cost per case successfully treated. This model uses input data on efficacy, compliance and treatment success without full compliance to estimate the average cost-effectiveness ratio for SP and AL. The input data has been gathered from two parallel surveys on drug efficacy and compliance as indicated earlier.

The model begins with a cohort of 55,509 patients presenting for treatment with SP and AL. The choice of 55,509 was a convenience as this was the total number of malaria cases recorded in 2005. As indicated above, using in vivo data on drug efficacy and compliance from the same population the curative success rate for both SP and AL is estimated. In particular, the model estimates the number of cases successfully treated from an equal cohort of 55,509 of patients under the two regimens, SP and AL, using the parameters contained in Table 2, according to the formula:

Number of cases successfully treated = *R*1* [(*R*2*R3) + (1 - *R*3)*R*4]

The first component on the left hand side measures the number of cases successfully after full compliance, while the second term measures the number of patients who are cured from the proportion that is assumed not to fully comply with the treatment dosage.

In this model, it assumed that 50% of patients who do not comply with the complete dosage of AL are still cured. A recent study assumed that 45% of non-complying patients using AL in similar settings still get cured [24]. Interviews with malaria case management experts at the National Malaria Centre also suggest that this is a realistic assumption.

As was shown in Table 1, the average cost per case treated (i.e. given anti-malarial) was about US$7.34 and US$6.19 for AL and SP, respectively. The study also estimates that malaria expenditure per capita on first-line treatment is higher if AL is used. This finding confirms unequivocally that the decision to adopt AL as the first-line drug for malaria treatment will require additional financing for malaria case management, at least in the short term (28-day window).

However, the average cost-effectiveness ratio, defined as the total treatment cost divided by the total number of cases successfully treated (i.e. cost per cured case), has been estimated at US$8.57 and US$10.65 for AL and SP respectively, as shown in Table 2. On this account, treatment with AL is more cost-effective than SP. This finding clearly indicates that as much as treatment with SP may appear cheaper in terms of budgetary outlays, it is shown that as the cost per the relevant output produced (cases cured) works out lower with AL. A crucial implication of this result is that for a given amount of resources (fixed case management budget), AL produces more cured patients while the use of SP would have given us more dosages dispensed but with proportionately less cured cases. According to the rule of thumb in cost-effectiveness analysis, the answer to the question of whether AL is cost-effective is in the affirmative.

### Incremental cost-effectiveness ratio (ICER)

The demonstrated superior effectiveness of AL has come at an additional price. This extra or incremental cost of producing one extra successful cure of an episode of malaria is known as the incremental cost-effectiveness ratio. Referring to the formula presented earlier, the incremental cost-effectiveness ratio of AL estimated in this study is US$4.10. This is the amount that it costs to achieve one extra successfully-treated case using AL in relation to SP. This estimate of incremental cost-effectiveness is within an order of magnitude of estimates from similar studies. A study in Tanzania estimated that the incremental cost of changing malaria first-line treatment from CQ to SP was US$0.20 [25].

### Incremental cost-effectiveness of AL including second line treatment

An incremental cost-effectiveness analysis of first line treatment might be considered incomplete particularly in the context of AL whose main impact, in this context, lies in reducing the cost of advanced malaria. ACTs are particularly favoured for early and more vigorous action against the parasite thereby reducing the probability of disease aggravation, mortality and recurrence of malaria. This section presents a simplified model that estimates the incremental cost-effectiveness ratio of using AL as first line treatment versus a regimen that uses SP for first line. In both cases, quinine is used as second line treatment of complicated or severe malaria. Based on data from all sites, the weighted average cost of severe malaria treatment was worked out to be US$ 16.32.

Results of the incremental cost-effectiveness ratio including the costs of repeated first-line and second-line treatments are constructed in table 3.

**Table 3:** Incremental cost-effectiveness of artemether-lumefantrine including second line treatment

	AL	SP
1. Total patients	55,509	55,509
2. First wave first-line, US$^a^	407,436.06	343,600.71
3. Second wave first-line, US$^b^	58,263.36	143,968.70
4. Second line treatment, US$^c^	6,088.60	182,575.57
5. Incremental cost, US$	-176,486.97	-
6. ACER including second line treatment, US$	9.92	20.78
*7. ICER including second line treatment, US$*	*-11.52*	-

(a) All patients receive first wave first-line

(b) 14.7% of AL and 41.9% of SP recipients fail first-line and receive repeated dosage

(c) cost of second line treatment is us$16.32

AL is dominant indicating that there are resource savings from reduced second line treatment that are associated with AL. The data shows that these savings emanate from comparatively fewer patients treated more than once with AL and even fewer patients treated for complicated malaria. These phenomena are known from a public health point of view but have not really been demonstrated in an economic framework. In this broader perspective, the case for AL is even more persuasive.

## Discussion

This study has revealed that adopting AL over SP raises the total cost of treatment thereby requiring substantial investments into the national malaria case management. However, the study has also demonstrated that AL produces much better health outcomes in terms of curative success and reduction in the cost of complicated or severe malaria treatment. Despite its higher cost per complete dosage, the cost per case treated successfully is much lower with AL on account of the effectiveness of AL in eradicating malaria infection in patients within the 28-day period. The cost-effectiveness ratio calculated in this study compare very well with similar findings from similar setting [22]. This reinforces the superiority of AL over SP from a cost-effectiveness perspective. In addition, it has been shown that a reduction in the burden of severe malaria reduces the need for hospital care. This leads to cost savings on reduced hospitalization. The health benefits of AL are likely to offer a considerable opportunity for reducing demands on scarce human resources given that malaria accounts for a highest proportion of visits and hospital admissions.

Further, a simple model applied in this study suggests that the incremental cost effectiveness ratio of AL falls well within customary incremental cost thresholds. In fact, by the recent standards of WHO, AL would fall within the category classified as highly cost-effective interventions. A policy decision is needed to evaluate whether this incremental cost-effectiveness ratio of US$4.10 is worth paying for. A natural perspective to take in evaluating this figure is to refer to the WHO Commission on Macroeconomics and Health criteria on incremental cost-effectiveness ratios (ICER) of US$25 or a nation's per capita Gross National Product [26,27].

The reduction in treatment failure and the prevalence of severe malaria in a cohort of patients that is associated with AL has naturally raised issues about causality. Using data obtained from facilities on cases of severe malaria cases recorded, it is indicated that a reduction of up to 91% was observed. This analysis excludes the confounding effects of reporting bias. If there is greater systematic under-reporting of severe malaria in population during the post-intervention period (in 2005) than was the case previously, then these results are likely to be biased upwards. The contribution of AL to this impact is still contentious. Malaria experts in Zambia have expressed an opinion that AL has contributed to a reduction in malaria morbidity. Collaborating data from an independent study point to a strong correlation between AL use and reduction in severe malaria admissions. In particular, at the Macha Malaria Institute in the southern province of Zambian, researchers show that cases of severe malaria in children had declined to virtually zero. Intrigued by dramatic reduction in malaria cases after the introduction of ACTs, the study team at Macha began to collect data on children with malaria.

"Malaria prevalence surveys are showing less than 5% parasite positivity rate by thick smear compared to 50% or more in previous years by this time in the rainy season. Interestingly, these surveys are showing that there is no decline in the number of anophelines."

Interviews with malaria experts introduced a useful albeit subjective dimension of explaining the contribution of AL to malaria morbidity and mortality trends. Opinions from field researchers in Macha suggest a strong link between AL and the decline in malaria morbidity and mortality. *"Thus, it seems to me, that while there should be no competition between the various interventions (we all need each other), that the evidence is much more strongly in favour of effective first-line therapy with ACTs than it is with use of ITNs or IRS programmes, if we want to decrease malaria morbidity and mortality" *[28].

"Admissions to the children's ward for malaria are still very rare so far this year as compared to previous years. I we have probably admitted only 14 cases between November 2005 and February 2006. Out of these, four were severe malaria cases. In the 2001–2 season, Macha hospital admitted 1,517 children with severe malaria and for a similar period in 2004–5, we recorded 157. Malaria case fatality rates decreased from 52 to 7."

The Macha Malaria Research Institute team believes that eliminating malaria among the population though effective case management has helped reduce the transmission rate within the population. Importantly, it should also be borne in mind that in the villages around Macha, there has been no in-door residual spraying (IRS) activities while the insecticide treated net (ITN) coverage is very low (assumed by Macha malaria institute staff to be at about 4–6%). Although these claims of AL effectiveness appear credible to clinicians and many field workers, they can only be validated by systematically gathered data. In addition, it remains to be seen if these observations are only short-term changes or they can be sustained over a longer period.

This study conducted interviews with health staff and community members through focus group discussions at three of the six sites (Chingola, Kabwe and Samfya). The information gathered indicated that public perceptions about the curative advantages of AL over previous drugs have seemingly helped to boost demand for public health care and helping to initiate early treatment. Though this information is anecdotal, the views from both health and community workers regarding the effectiveness of AL were unanimous. A community worker at a focus group discussion in Kabwe giving her views on the positive influence of an effective drug on choice of care:

"The experience with patients who have been put on artemether-lumefantrine has shown that many patients are responding well to this drug and seem to prefer it. Even patients that used to go to witch doctors are now reporting to public facilities for treatment. In the past when people did not get cured with a single visit to a clinic, they would turn to a traditional healer."

Further, the impact of AL on the scale estimated in this study is in line with findings from at least two surveys that have evaluated the efficacy of AL in Africa as well as another independent study conducted in Zambia [29-33]. Finally, it is important to remember that the potential contribution of malaria prevention interventions such as ITNs and IRS on observed the cost-effectiveness of AL is likely to be very limited because of their low coverage. Many malaria experts have considered that for ITN and IRS interventions to make a substantial impact on malaria incidence, population coverage should be at least 50%.

### Sensitivity analysis

Sensitivity analysis was performed to show whether the parameters used in estimating cost-effectiveness ratios are robust to some degree of uncertainty. Importantly, this analysis allows an evaluation of the potential effect of misdiagnosis in the context of the design of this study. For several reasons, malaria misdiagnosis is perhaps a rule rather than an exception in many endemic countries. Assuming that up to 60% of cases recorded and treated as malaria might not have been true malaria, the cost and cost-effectiveness implications are considered in table 4. Basically, both the ACER and the ICER of AL rise significantly, indicating that presumptive treatment of fevers reduces the attractiveness (i.e. cost-effectiveness) of AL in relation to SP. The cost-effectiveness implications of alterations to the values of other parameters applied in estimating ACER and ICER are shown in table 4. The main conclusions remain as before, although the ICER increases but still remains in the same order of magnitude. Thus, the results of this sensitivity analysis suggest that the key cost-effectiveness results remain robust to plausible variations of the main assumptions used in the model.

**Table 4:** Sensitivity of CEA results to parameter alterations

*Parameter alterations*	*Effect on ACER and ICER*	*CEA conclusion*
Discount rate in estimating capital costs is 10% instead of 3%	No effect as cost of both AL and SP rise by same margin	Unchanged
AL costs 10% more than estimated	ACER increases to US$8.67 ICER increases to US$4.57	Unchanged in terms of ACER but ICER increases by 18%
AL costs 15% more than estimated	ACER increases to US$8.75 ICER to US$4.81	Unchanged in terms of ACER but ICER increases by nearly 20%
AL costs 50% more than estimated	ACER and ICER of AL increase to US$9.29 and US$6.47	Unchanged in terms of ACER but ICER increases by 27%
Compliance of AL is lower at 65%	ACER increases to US$9.02 ICER increases to US$4.95	Unchanged but ICER increases by
Efficacy of AL is lower at 90%	ACER increases to US$9.17 ICER to US$5.25	Unchanged but ICER increases by nearly 30%
Efficacy of AL is lower at 80%	ACER increases to US$10.12 ICER increases to US$7.99	Unchanged but ICER increases by 95%
Proportion of patients seeking second line treatment is 4.7% in both AL and SP	ICER of AL is now US$3.10	The cost of additional successfully treated case is US$3.10
Diagnosis of malaria is poor and 60% of cases treated are actually non-malaria cases for both AL and SP	Incremental cost will remain the same but the number of cases successfully treated will drop. The cost per cases successfully treated rises proportionately to US$ 21.29 and US$26.62 for AL and SP respectively. ICER rises from US$4.10 to US$10.25.	Decision still in favour of AL but the costs are much higher with ICER more-than doubling
Proportion of patients progressing to severe malaria is same at 4.7% for both AL and SP	ACER of SP decreases substantially from US$20.78 to $15.75 but still higher in relation to AL. ICER increases from US$-11.52 to US$-0.94.	ACER and ICER conclusions remain same

### Limitations and recommendations for further research

A number of limitations which raise some caveats of this study can be mentioned. One limitation of the study is that it did not consider a societal perspective of costing. It would be of great interest to see how resilient the cost-effectiveness conclusions derived from this perspective are, to the inclusion of societal, or at least patient, costs. Second, the survey conducted in six of the sentinel sites that have witnessed anti-malaria activities for some time, the results may not be generalized to the entire population. It would be interesting to see what future work in other areas could suggest. Third, the study adopted a definition of malaria imposed by actual behaviour of health workers. This may have interfered with the estimated cost-effectiveness ratios in one way or the other. It would be important to study the effects of adherence to diagnostic results especially in the case of adult patients. Future work could focus on this important issue.

Further, this study considered only treatment success within a 28-day period. Cases that were reported after this period were considered to be new infections. In addition, it remains to be seen if the impact observed with the introduction of AL will be sustained over a long time. Since resistance and treatment failure are dynamic concepts, the cost-effectiveness analysis of introducing a new treatment such as ACTs should not be done from a static point of view but rather from a dynamic perspective.

## Conclusion

This study is one of pioneering studies investigating the cost-effectiveness of AL in a real African health system setting. Concerns over the high cost of ACTs relative to SP and chloroquine have dominated the policy debate around the introduction of ACTs in Zambia and other low-income countries. Many commentators have questioned both the affordability and sustainability of ACTs in a resource-poor and weak health system setting such as Zambia's. A relevant question to ask, however, is whether the extra cost of AL is worth the extra health benefits that AL confers on patients and the health system in general. Accordingly, this study has performed a cost-effectiveness analysis to provide guidance on the key issues arising from changing malaria treatment policy in the face of drug resistance to other anti-malarials. By comparing both the health effects and cost of AL against SP, a cost-effectiveness framework helps us to inform this debate through a careful assessment of both costs or financial outlays and health benefits.

As the debate on affordability of AL continues, this study shows that substantial extra resources will be required to sustain the scale up of treatment with AL as well for a supporting prevention strategy. Cost-effectiveness analysis should not be confused with costing analysis or cost-benefit analysis. While it is acknowledged that implementing national ACT program will require considerable resources in the short-term, the thrust of this study demonstrates that the health gains (as defined by treatment success) from every dollar spent are significantly greater if AL is used rather than SP. This is a different issue from stating that AL is affordable. In fact it was shown that for a limited budget, SP will provide more dosages, though with much less positive effect.

The study points out that case management alone is insufficient to guarantee a sustained reduction in morbidity and mortality. While AL is cost-effective, it is not a subject for transmission control through bed nets and residual spraying. A combination of prevention to halt malaria transmission and the use of an effective drug for prompt treatment of cases would confer substantial economic and health gains. In conclusion, this analysis strongly suggests the decision to adopt AL is clearly justifiable on both economic and public health grounds.

## Competing interests

The authors would like to acknowledge that Norvatis Pharmaceuticals provided the funding for this study through the Zambian Ministry of Health. However, none of the authors works for, or represents in any way, Norvatis Pharmaceuticals.

## Authors' contributions

PC participated in developing the research protocol, field work supervision, data analysis and drafting this manuscript. FM was involved in designing data collection instruments, field supervision, data analysis and drafting this manuscript. BMC was involved in designing the research protocol, data collection, data analysis and drafting this manuscript. PB was involved in research protocol development, data collection, supervision of field work and editing this manuscript. MH was involved in research protocol development, data collection, supervision of field work and editing this manuscript. NS participated in designing the research protocol, data collection, supervision of field work, data analysis and drafting this manuscript. TO reviewed the methodology, undertook some field visits and participated in the drafting of the manuscript.
